# Experimental and theoretical quantum chemical studies of 2-(2-acetamidophenyl)-2-oxo-*N*-(pyridin-2-ylmethyl)acetamide and its copper(II) complex: molecular docking simulation of the designed coordinated ligand with insulin-like growth factor-1 receptor (IGF-1R)

**DOI:** 10.1186/s13065-024-01217-z

**Published:** 2024-06-13

**Authors:** Doaa S. El-Sayed, Leena Sinha, Amina A. Soayed

**Affiliations:** 1 Chemistry Department, Faculty of Science, Alexandria University, P.O. Box 426, Ibrahimia, 21321 Alexandria Egypt; 2 Department of Physics, University of Lucknow, Lucknow, India

**Keywords:** 2-(2-Acetamidophenyl)-2-oxo-*N*-(pyridin-2-ylmethyl)acetamide, DFT, ESR, ^1^H-NMR

## Abstract

Newly synthesized ligand 2-(2- acetamidophenyl)-2-oxo-*N*-(pyridin-2-ylmethyl)acetamide and its copper(II) complex were characterized by elemental analyses, FT-IR, UV–Vis., ESR, ^1^H-NMR, and thermal analysis along with the theoretical quantum chemical studies. Combined experimental and theoretical DFT (density functional theory) studies showed the ligand to be a tridentate ligand with three coordinate bonds. The complex was suggested to be in a distorted octahedral structure with d_x_^2^_-y_^2^ ground state. The activation energy, ΔE^*^; entropy ΔS^*^; enthalpy ΔH^*^ and order of reaction has been derived from differential thermogravimetric (DTA) curve, using Horowitz–Metzeger method. The nujol mull electronic spectrum of the ligand and Cu(II) complex have been recorded and the difference of the excited and ground state densities has also been theoretically calculated and plotted to investigate the movement of electrons on excitation. The Cu(II) complex was evaluated for its antibacterial activity against two bacterial species, namely Escherichia coli (E. coli) and Staphylococcus aureus (S. aureus). Antifungal screening was performed against two species (Condida albicans and Aspergillus flavus). The complex under investigation was found to possess notable biological activity. Molecular docking investigation predicted different types of non-covalent interactions of the synthesized ligand towards Insulin-like growth factor 1 receptor (ID: 5FXR).

## Introduction

Currently, the preparation of new copper complexes and investigation of their biological activities for pharmaceutical applications is one of the most dynamic fields. Copper(II) complexes have been found to be more potent than their parent ligands in biological activity [1–4] as well as in industrial applications [5, 6]. The importance of mononuclear copper complexes also lies in the fact that these can possibly mime the active sites of metalloproteins such as the enzyme galactose oxidase [7] and nitrite reductase [8]. The structural arrangement of the complexes formed can be related to their individual molecular species and to the type and/or position of different substituents in the ligand and metal ions. A deeper understanding of the roles played by pyridine candidates in biological systems may result from the synthesis of their metal complexes. Like these ligand compounds have a wide range of medicinal applications and can coordinate with transition metal ions. Additionally, the development of new metal-based chemotherapeutic agents may be aided by these investigations.

Complexes of di- and polypyridyl ligands with transition metal ions have attracted great attention because of the coordination versatility of such ligands as well as their potential application in a number of biological, catalytic, photoactive and sensor applications [9, 10]. Picolylamine (2-Amino-methylpyridine) derivatives are known as useful chelating ligands for many metal ions. These ligands have become important for their bioinorganic and medicinal purposes [11, 12], and they may bind meridional or vicinal to a metal. Picolylamine can act as a bridging ligand as well [13] but it is known that the bridging mode of chelation of picolylamine is rare in case of copper (II) complexes [14]. The interaction of Cu(II) with various picolylamine derivatives containing substitution at the secondary nitrogen center has also been widely investigated [15–25], a number of such complexes are effective reagents for the interaction and the oxidative cleavage of DNA [26–32]. In order to mediate the direct contact of the metal center with the substrate, the mimetic complexes’ reactivity exhibits dependency on the existence of either labile ligands or “open” coordination sites [33].

Previously, the reaction of N-acetylisatin with some alcohols, different amines and diamine, pyrrolidine and water yielded products resulting from nucleophilic attack at the C-2 carbonyl which led to the heterocyclic ring cleavage [34, 35].

The biological properties of pharmaceutical compounds are significantly influenced by the C-N bond, which is an essential and widely occurring structural motif found in thousands of natural products. In more details, cleaving activated amides selectively at the C-N bond has proven to be an effective and vital method for obtaining a variety of compounds. Recently, significant advancements were achieved and a number of methods for amide activation and C–N bond cleavage were described [36, 37] making amides interesting synthons in organic synthesis. Activated amides are novel acyl or aryl resources that are more effective than traditional acylation reagents like acid anhydride and acyl chloride.

In the present work, we report the synthesis and study of physicochemical and biological activities of of 2-(2-acetamido-phenyl)-2-oxo-*N*-(pyridin-2-ylmethyl)acetamide and its copper (II) complex. The reaction of N-acetylisatin with 2-aminomethyl pyridine results in the cleavage of heterocyclic ring and lead to the production of 2-(2-acetamido-phenyl)-2-oxo-*N*-(pyridin-2-ylmethyl)acetamide. Characteristic investigation was performed and computational study via DFT were applied to support the structural and electronic behavior of the synthesized complex. Antimicrobial activity of the studied compound was screened and molecular docking perturbation support the biological behavior.

## Experimental

### Chemicals used

All chemicals and solvents were obtained from Sigma–Aldrich Chemical Company and used as received.

### Synthesis of 2-(2-acetamidophenyl)-2-oxo-*N*-(pyridin-2-ylmethyl)acetamide

#### Conventional procedure

To the solution of *N*-acetylisatin (10 mmol) in acetonitrile (20 mL), pyridin-2-yl-methanamine (10 mmol) was added at room temperature. The reaction mixture was stirred at room temperature for 12 h. The solvent was removed next day under vacuum and the crude product was recrystallized from dichloromethane and hexane (1:2) to obtain the pure product.

#### Procedure for microwave-irradiation

Employing a multimode reactor (Synthos 3000, Aton Paar GmbH, 1400W maximum magnetron); the initial step was conducted with a Teflon vessel rotor (MF 100). The vessel contained *N*- acetylisation mixed with pyridin-2-ylmethanamine in small amount of acetonitrile (2–5 mL). The vessel was purged with nitrogen gas for 5 min and then was placed in the corresponding rotor, fixed by screwing down the upper rotor place, and finally the rotor was closed with a protective hood. The vessel was heated for 3 min. at 80 °C and held at the same temperature for a further 2 min (~ 2 bar pressure, 200 W). Cooling was accomplished by a fan (5 min). The final product was dried, and recrystallized from dichloromethane-hexane (1:2).

The product was obtained as light beige crystals; m.p. 142–143 °C; IR peaks (cm^−1^): 3489 (NH + H-bonded NH), 1744 (C=O), 1697,1628 (C=O amide), 1597 (C=N py). ^1^H-NMR (DMSO-d6): δ 2.00 (s, 3H, COCH_3_), 4.491, 4.503 (m, 2H, CH_2_), 7.215–7.793 (m, 4H, ArH), 8.502, 8.511 (m, 4H, py), 9.251, 9.263, 9.275 (m, 1H, NHCOCH_3_), 10.568 (s, 1H, NHCH_2_).

### Preparation of the Cu(II) complex

**Scheme 1:**
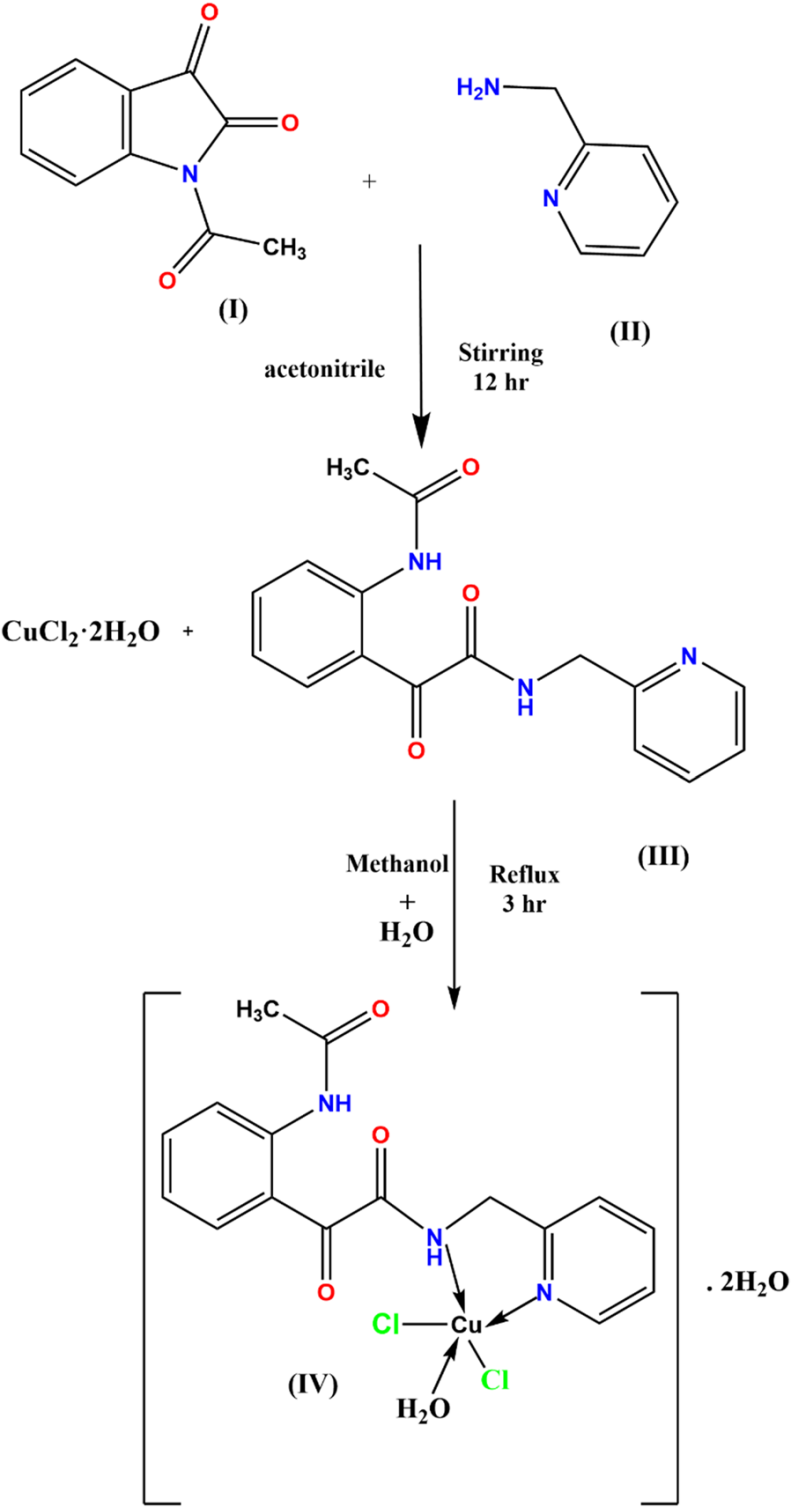
Synthesis of Cu(II) complex. (I) N-acetylisatin, (II) pyridin-2-yl-methanamine, (III) 2-(2-acetamidophenyl)-2-oxo-N-(pyridin-2-ylmethyl)acetamide, (IV) [Cu.L.Cl_2_.H_2_O].2H_2_O

The Cu(II) complex was prepared by adding a solution of CuCl_2_·2H_2_O (0.8524 g, 5 mmol, in 25 mL methanol) to a hot 50 mL methanol solution of the organic ligand, (5 mmol). This solution was refluxed for 3 h. After that, the resulting solution was allowed to evaporate at room temperature to give the turquoise blue Cu(II) complex, which was collected and washed with cold methanol. The product was dried over anhydrous calcium chloride in a desiccator (~ 70% yield). However, our attempts to isolate single crystals suitable for X-ray crystal structure determination were not successful. Thus, theoretical DFT studies were done in order to elucidate the structure of the ligand and complex under study. Scheme 1 displays the synthetic pathway of the studied Cu(II) complex.

The analytical data showed that the turquoise blue complex is mononuclear with a 1:1 (M:L) mole ratio. Molecular formula C_16_ H_21_ O_6_ N_3_ Cl_2_ Cu, formula weight (485.805); %C: 39.558 (found: 39.627%), %H: 4.359 (found: 4.122%); %N: 8.649 (found: 8.876%), %O: 19.759 (found: 20.011%), %Cu: 13.079 (found: 13.326). m.p. ˃ 300 °C; IR (cm^−1^): 3489 (NH + H-bonded NH), 1744 (C=O), 1697,1628 (C=O amide), 1597 (C=N py). ^1^H-NMR (DMSO-d6): δ (ppm) 1.880 (s, 3H, COCH_3_), 4.581 (m, 2H, CH_2_), 7.051–7.912 (t, 4H, ArH), 8.077, 8.928 (m, 4H, py), 9.323, 9.709 (m, 1H, NHCOCH_3_), 10.480, 10.599 (s, 1H, NHCH_2_).

### Physicochemical studies

The elemental analyzer of type 240 Perkin Elmer, was used to perform (C, H and N) elemental analyses. Additionally, content of metal ion was measured using the computer controlled atomic absorption spectrophotometer of type 850-Fisher Jarrell-Ash [1]. IR spectra of the studied ligand and its Cu complex were recorded with a Perkin-Elmer FT-IR of type 1650 spectrophotometer in region of wavenumber 4000–400 cm^-1^. ^1^H-NMR spectra were studied using the solvent DMSO-d6 (Merck). The thermal analyses curves were performed using thermal analyzer in platinum crucibles. UV–Vis spectra were studied in range 200-1000 nm. Magnetic properties, involving molar magnetic susceptibility, was measured at 298 K. ESR spectra were studied at room temperature (298 K). All experimental measurements were determined using fresh prepared solutions.

### Biological activity

Antimicrobial activity of the Cu(II) complex was conducted using a modeled Kirby–Bauer disc method of diffusion [38]. Practically, growing a certain volume of tested bacteria or fungi (100 µL) fresh media (10 ml) was initiated to reach about 108 cells / mL bacterial growth or 105 cells / mL fungi growth [39]. A definite volume of microbial suspension (100 µL) was used for spreading into plates of agar. The Plates were prepared with filamentous fungi such A. flavus at room temperature(25 °C) for 48 h; Staphylococcus aureus (Gram +) bacteria, Escherichia coli (Gram −) bacteria at 35 to 37 °C for 24 to 48 h, and C. Albicans, as yeast, incubated at 30 °C from 24 to 48 h. The inhibition zone diameters were measured in millimeters [38].

### Computational methodology

Density functional theory [40] with hybrid Becke’s three parameters and the Lee–Yang–Parr functional (B3LYP) [41–43] using LANL2DZ basis set for the Cu metal centers and 6-31G (d) basis set for the H, C, N and O atoms in the gas phase has been employed in the present investigation [44–46]. Full geometry optimizations and vibrational analyses have been performed using the Gaussian 09 software [47]. DFT methods are recurrently used in modelling metal complexes and have good experimental consonance with the infrared frequencies. Gauss-view 5 molecular visualization software with the VEDA 4 software [48] have been used for the vibrational assignments of the normal modes. Theoretical investigation of the electronic structure of the ligand and its metal complex have also been carried out and the highest occupied molecular orbital (HOMO) and lowest unoccupied molecular orbital (LUMO) have been plotted at the same level of theory.

### Molecular docking performance

The potent docking pose was predicted for the studied ligand using autodock 4.2. software [49] for conformational calculations and studio discovery (http://www.accelrys.com) program for docking visualization. The protein target was selected for Insulin-like growth factor 1 receptor (ID: 5FXR). The receptors were prepared by removing any ions, water molecules, small ligands and the addition of polar hydrogens [50]. The crystal structures of the bacterial proteins were obtained from the protein data bank (http://www.rcsb.org./pdb). Initial preparation steps were considered before the docking process started, such as water and hetatoms removal from protein crystallographic structure. additionally, charge adjustment and polar hydrogen inclusion for getting the protein and ligands ready in the right format. The expected active spots were identified and the grid box dimensions were created. The grid box size was approximated with dimensions of 64 × 72 × 60 (Å^3^), 0.375 Å spacing, and grid centers x, y, and z of 18.822, 2.975 and 49.895, respectively. Genetic Algorithm (LGA) was thought to be the binding affinity mechanism [51, 52].

## Results and discussion

**Fig. 1:**
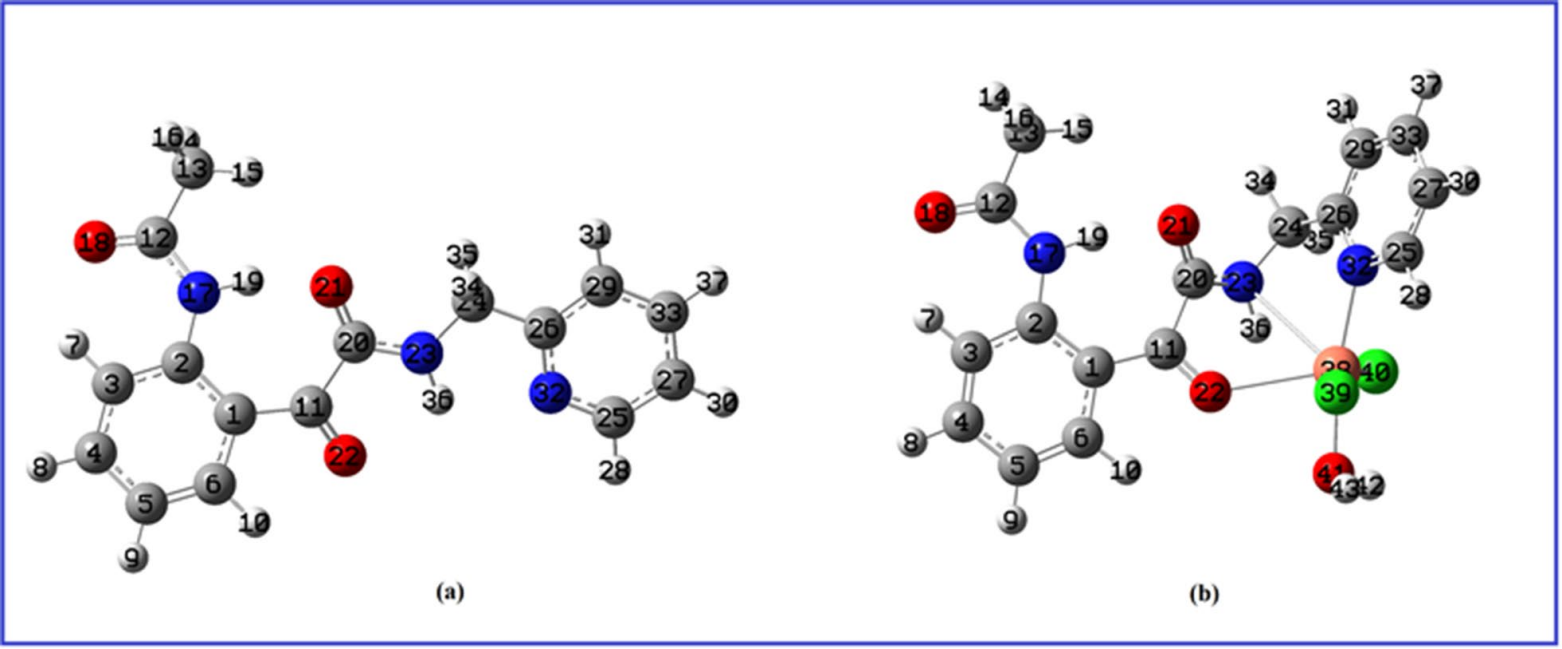
Optimized structure (**a**) Ligand (**b**) Cu(II) complex

The structures of the newly prepared 2-(2-acetamidophenyl)-2-oxo-*N*-(pyridin-2-ylmethyl)-acetamide ligand (L) and its [Cu.L.Cl_2_.H_2_O].2H_2_O complex were studied experimentally using elemental analyses, IR, ^1^H-NMR, UV–Vis. spectra, thermal analysis, magnetic moment for the copper (II) complex and theoretically using the density functional theory. In the absence of the X-ray crystal structure data of the ligand and its complex, quantum chemical calculations were employed to obtain the ground state optimized structure of the ligand (L) and its complex ([Cu.L.Cl_2_.H_2_O].2H_2_O) (Fig. 1).

### Geometry

The structure of the ligand as well as its Cu(II) complex were fully optimized using density functional theory. The stability of both the structures was verified by all positive wave-numbers from the output of frequency calculation. The ground state energies for ligand and the complex have been calculated to be − 1008.69854 a.u. and − 2201.48603 a.u., respectively. The optimized parameters of ligand and the complex are collected in Table 1. The [Cu.L.Cl_2_.H_2_O].2H_2_O complex was formed by the binding of the Cu metal ion to the N atom of the pyridine ring and the N atom of the CH_2_NHC=O group forming a five membered ring. Another five membered ring is formed by the binding of Cu ion to O22 and N23. The dihedral C11-C20-N23-C24 at − 152.9° in the ligand is changed to − 161.4° in [Cu.L.Cl_2_.H_2_O].2H_2_O. The dihedral C20-N23-C24-C26 which determines the orientation of pyridine ring with the amide (C20=O21-N23-H36) group in the metal complex is calculated at 67.0°, whereas the pyridine ring is almost planar in the ligand. The bond length of three C20=O21, C11=O22 and C12=O18 bonds in the ligand/Complex are calculated at 1.240/1.235, 1.227/1.236, 1.224/1.222 Å.

**Table 1:** Optimized parameters of the Ligand and its Cu(II) complex

Optimized parameters of ligand						Optimized parameters of [CuLCl_2_H_2_O].2H_2_O					
Bond Length in Å		Bond Angle in degrees		Dihedral Angles in degrees		Bond Length in Å		Bond Angles in degrees		Dihedral in degrees	
C1-C2	1.431	C13-C12-N17	113.4	H16-C13-C12-O18	62.0	C1-C2	1.433	C1-C2-N17	121.0	C3-C2- N17-C12	− 9.7
C2-C3	1.410	C13-C12-O18	121.2	H16-C13-C12-N17	118.2	C1-C11	1.479	C3-C2-N17	120.1	C1-C11-C20-O21	49.5
C11-C1	1.496	C12-N17-H19	116.6	H14-C13-C12-O18	− 54.3	C2-N17	1.394	C1-C6-H10	117.1	C1-C11-O22-Cu38	− 167.1
C12-N17	1.381	C12-N17-C2	128.8	H14-C13-C12-N17	125.5	C6-H10	1.084	C1-C11-O22	121.3	C1-C11-C20-O21	49.5
C12-O18	1.224	C1-C2-C3	118.7	H15-C13-C12-O18	− 176.3	C11-C20	1.546	C13-C12-N17	113.2	C1-C11-C20-N23	− 137.5
C12-C13	1.523	C2-N17-H19	114.4	O18-C12-N17-H19	− 176.9	C11-O22	1.236	C13-C12-O18	121.8	C2-C1-C6-C5	2.3
C13-H14	1.094	N17-C2-C3	120.2	O18-C12-N17-C2	− 2.1	C12-N17	1.388	N17-C12-O18	125.1	C11-C1-C2-N17	− 8.2
C13-H15	1.093	N17-C2-1C	12.1	H19-N17-C2-C1	− 19.8	C12-O18	1.222	C2-N17-C12	129.0	C11-O22-Cu38- O41	− 172.8
C13-H16	1.095	C2-C3-H7	118.0	H19-N17-C2-C3	160.8	N17-H19	1.022	C2-N17-H19	115.7	C13-C12-N17-C2	177.2
N17-H19	1.025	H9-C5-C6	120.3	N17-C2-C3-H7	1.6	C20-O21	1.235	C12-N17-H19	115.3	O18-C12-N17-C2	− 1.8
C11-O22	1.227	C5-C6-H10	120.2	N17-C2-C1-C11	− 5.7	C20-N23	1.351	N17-H19-O21	159.4	C20-C11-O22-Cu38	14.3
C11-C20	1.561	H10-C6-C1	117.2	N17-C2-C1-C6	179.3	O22-Cu38	2.554	C11-C20-O21	124.7	O22-C11-C20-O21	− 132.0
C20-O21	1.240	C6-C1-C11	112.2	N17-C2-C3-C4	− 178.8	N23-C24	1.446	C11-C20-N23	112.1	O22-C11-C20-N23	41.1
C20-N23	1.337	C2-C1-C11	129.7	N17-C2-C1-C11	− 5.7	N23-H36	1.015	O21-C20-N23	122.9	C25-N32-C26-C24	177.3
N23-H36	1.018	C1-C11-O22	119.8	C3-C2-C1-C11	173.7	N23-Cu38	2.984	C11-O22-Cu38	126.7	Cu38-O22-C11-C1	− 167.1
N23-C24	1.445	C1-C11-C20	125.6	C6-C1-C11-O22	19.9	C25-N32	1.350	C20-N23-C24	121.4		
C24-C26	1.518	O22-C11-C20	114.5	C2-C1-C11-C20	− 155.4	C26-C29	1.397	C20-N23-H36	119.4		
C26-N32	1.337	C11-C20-O21	125.8	C1-C11-C20-O21	− 4.3	C26-N32	1.350	C20-N23-Cu38	97.2		
N32-C25	1.340	O21-C20-N23	122.5	C6-C1-C11-C20	− 155.2	N32-Cu38	2.110	C24-N23-H36	116.7		
C29-C26	1.400	C11-C20-N23	111.7	O22-C11-C20-N23	− 1.2	Cu38-Cl39	2.327	C24-N23-Cu38	96.3		
		C20-N23-C24	122.1	C1-C11-C20-N23	174.1	Cu38-Cl40	2.343	N23-C24-C26	114.5		
		C20-N23-H36	119.7	O21-C20-C11-O22	− 179.6	Cu38-O41	2.060	C25-N32-C26	118.7		
		H28-C25-N32	115.9	O21-C20-N23-H36	179.0	O41-H42	0.975	C25-N32-Cu38	112.9		
		H28-C25-N32	115.9	C11-C20-N23-H36	0.6			C26-N32-Cu38	128.1		
		H28-C25-N32	115.9	O21-C20-N23-C24	0.0			C27-C33-C29	118.5		
		H28-C25-N32	115.9	C20-N23-C24-H35	− 58.2			C29-C33-H37	120.5		
		H28-C25-N32	115.9	C20-N23-C24-H34	57.9			O22-Cu38-N23	56.7		
				H36-N23-C24-C26	0.8			O22-Cu38-N32	111.4		
				C20-N23-C24-C26	179.8			O22-Cu38-Cl39	91.3		
				N23-C24-C26-N32	− 0.6			O22-Cu38-Cl40	105.2		
				H34-C24-C26-N32	121.2			N23-Cu38-N32	66.8		
				H34-C24-C26-C29	− 58.8			N23-Cu38-Cl39	128.1		
								N23-Cu38-Cl40	74.1		
								N23-Cu38-O41	120.7		
								N32-Cu38-O41	170.1		
								Cl39-Cu38-Cl40	157.7		

### FTIR spectral analysis

**Fig. 2:**
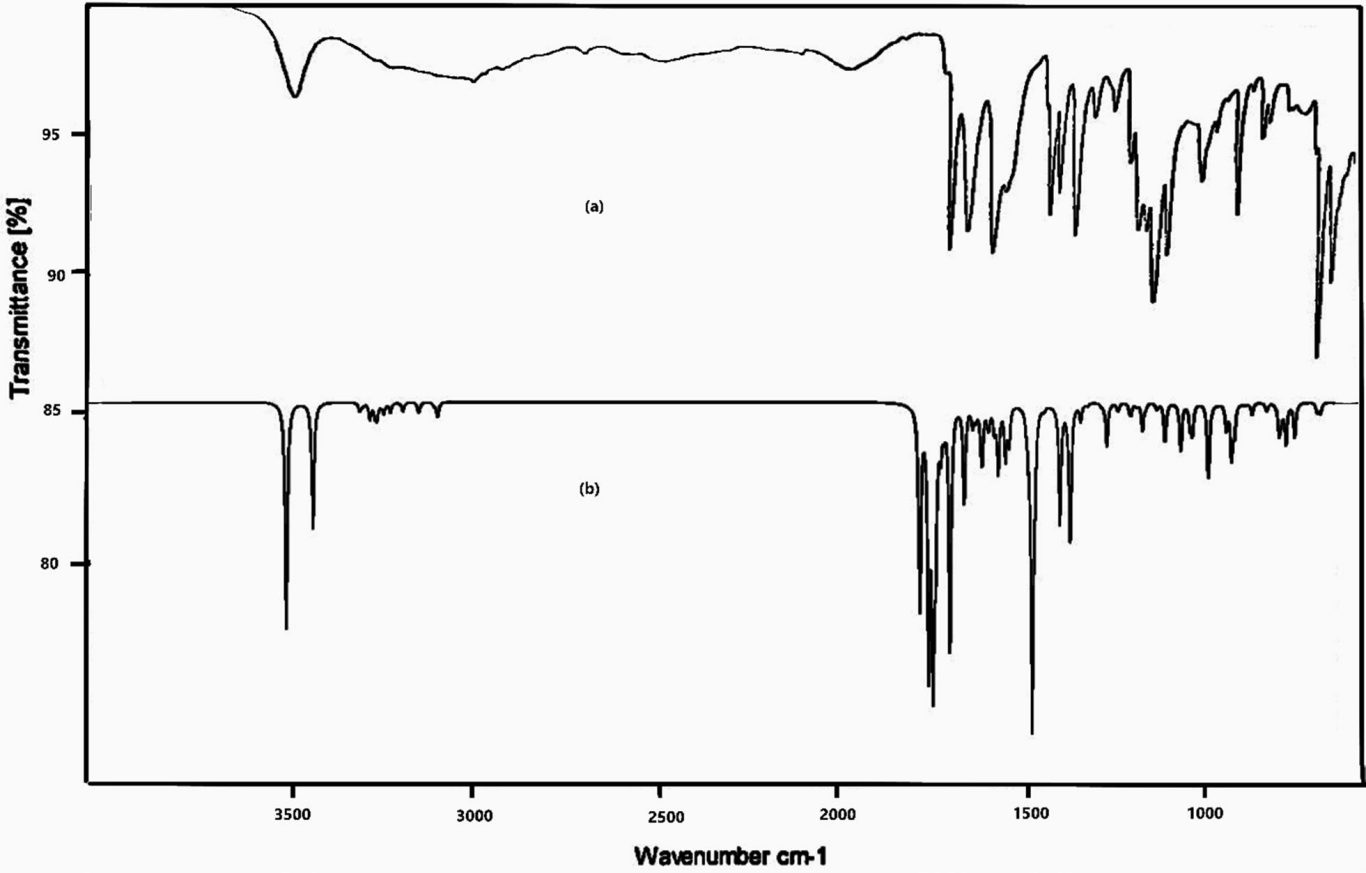
**a** Experimental FT-IR and **b** computational (DFT/B3LYP) IR spectra of the ligand

**Fig. 3:**
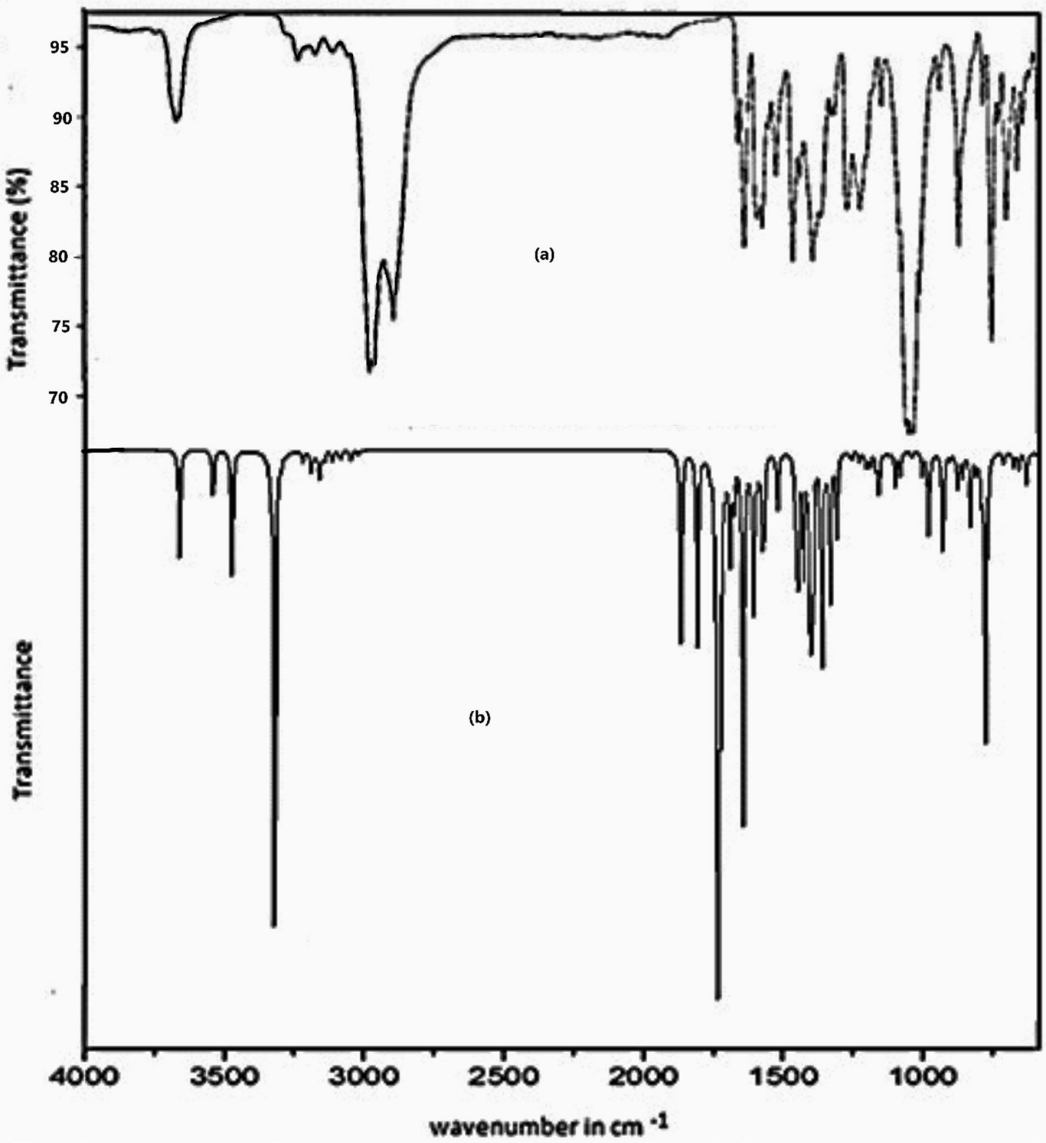
**a** Experimental FT-IR and **b** computational (DFT/B3LYP) IR spectra of the Cu(II) complex

The infrared spectra (FTIR) of the organic ligand and [Cu.L.Cl_2_.H_2_O].2H_2_O complex were analyzed and the proposed assignments of the bands are shown in Table 2. Experimental and simulated spectra for the ligand and the complex are given in Figs. 2 and 3 respectively. The N–H band of amines and amides absorb in the same region as O–H bond, but due to the greater electronegativity of oxygen than nitrogen, the O–H stretching band is much stronger than the N–H stretching band. N–H peaks usually are sharper, but weaker peaks than O–H bands [53]. The wavenumbers corresponding to three C=O bonds [54] in the ligand are calculated at 1735, 1687 and 1602 cm^−1^ with experimental values at 1744, 1697 and 1597 cm^−1^. The infrared spectral bands due to –NH groups in Cu complex, are shifted to lower frequencies at 3240 and 3179 cm^−1^ as compared to the NH bands of ligand (3480 and 3240 cm^−1^) either due to the participation in complex formation or involvement in H-bonding with a C=O group. One C=O band out of three bands in the ligand, disappeared completely in the Cu(II) complex indicating its participation in complex formation. The bands at 1697 and 1597 attributed to C=O amide I and II were blue shifted (1673 and 1585 cm^−1^) as a result of complex formation and H-bonding with -NH group. A new band appeared at 3672 cm^−1^ as a result of the presence of a coordinated water molecule [54] and / or H-bonded –NH group [55]. The characteristic pyridine stretching mode having contribution from C=C as well as C=N stretch at 1628 cm^−1^ shifted to 1606 cm^−1^ due to its contribution in complex formation. This is corroborated by the increase in C26-N32 and C25-N32 bond lengths on complexation. The CH_2_ scissoring calculated at 1470 cm^−1^ (L) is in good agreement with peak at 1476 cm^−1^ in IR spectrum. C12-N17 stretching observed and calculated at 1310 and 1301 cm^−1^ respectively is a mixed mode with contribution from C–C stretch and C–C–C bending of phenyl ring. Methylene C_24_H_2_ stretching being a part of five membered ring in the metal complex shows strong IR peaks on complexation at 2987 and 2972 cm^−1^ in comparison to the weak CH_2_ stretching vibration in the ligand at slightly lower wavenumbers. New bands at 656 and 677 cm^−1^ were observed corresponding to the stretching vibration of M–O and M–N, respectively [56].

**Table 2:** Selected vibrational modes of the ligand and its Cu (II) complex

vibrational mode	Ligand		Complex	
	Exp. wavenumber	Theoretical wavenumber	Exp. wavenumber	Theoretical wavenumber
υ (N23-H36)	3489	3423	3240	3440
υ (N17-H19)	3240	3224	3179	3280
υ (C24H_2_)	2932, 2919	2957, 2937	2987, 2972	3040, 2994
υ (C=O)	1744, 1697, 1597	1735, 1687, 1602	16,73, 1585	1692, 1684
υ (C=N + C=C) characteristic Pyridine ring	1628	1664	1606	1638
C24-N23 stretch	1159	1116	1066	1074
C24H_2_ scissoring	1476	1470	1451	1436
C13H_3_ asymmetric bending	1453	1470	1477	1455
C13H_3_ symmetric umbrella bending	1363	1383	1406	1381
υ (C12-N17) + υ CC Ph ring + CCC bend Ph ring	1310	1301	1310	1304
Pyridine trigonal bend	969	984	1027	1000
Cu38-N23-H36 bend	–		717	713

### ^1^H-NMR spectral analysis

**Fig. 4.**
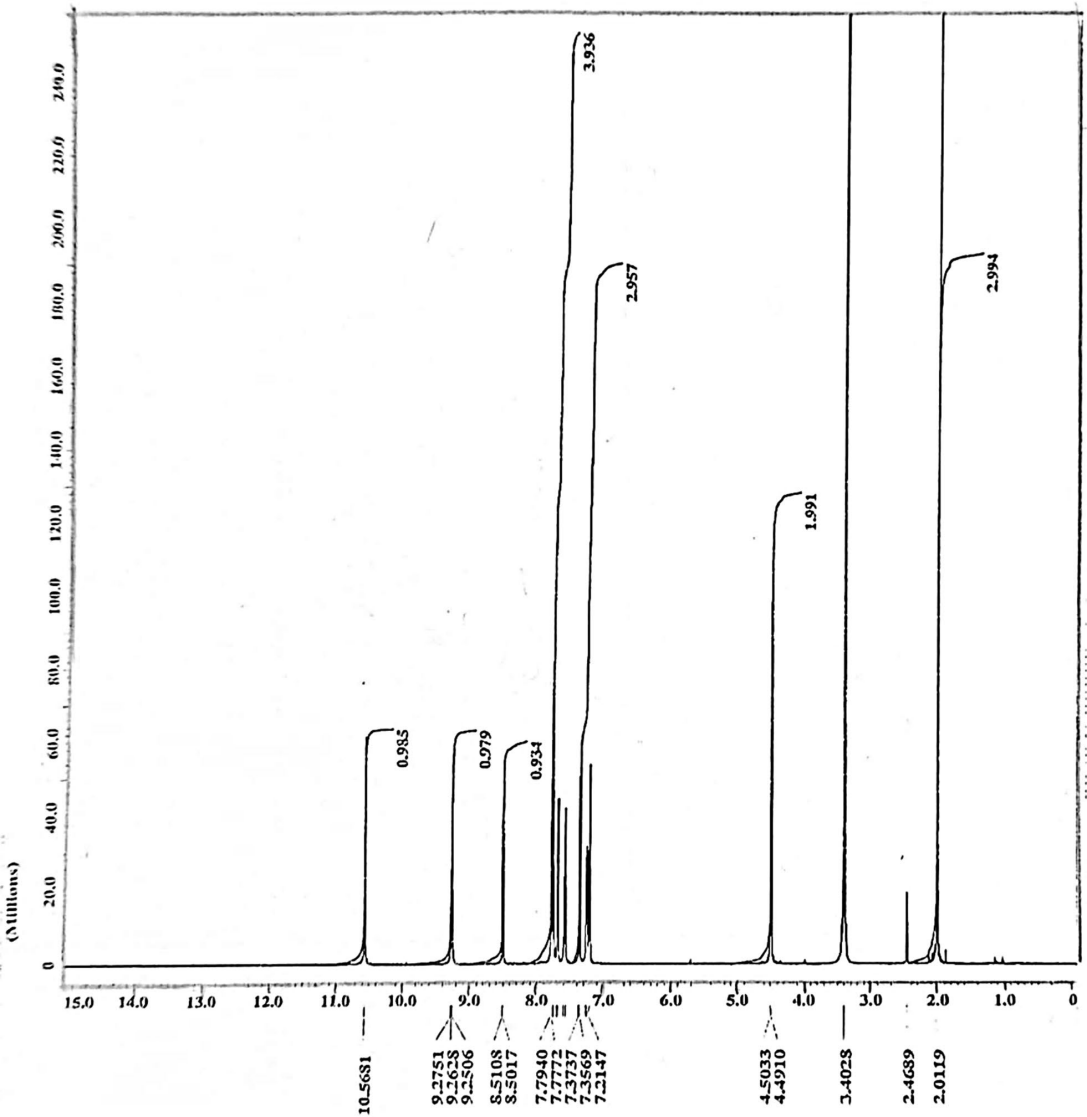
^1^H-NMR spectrum of the ligand

**Fig. 5.**
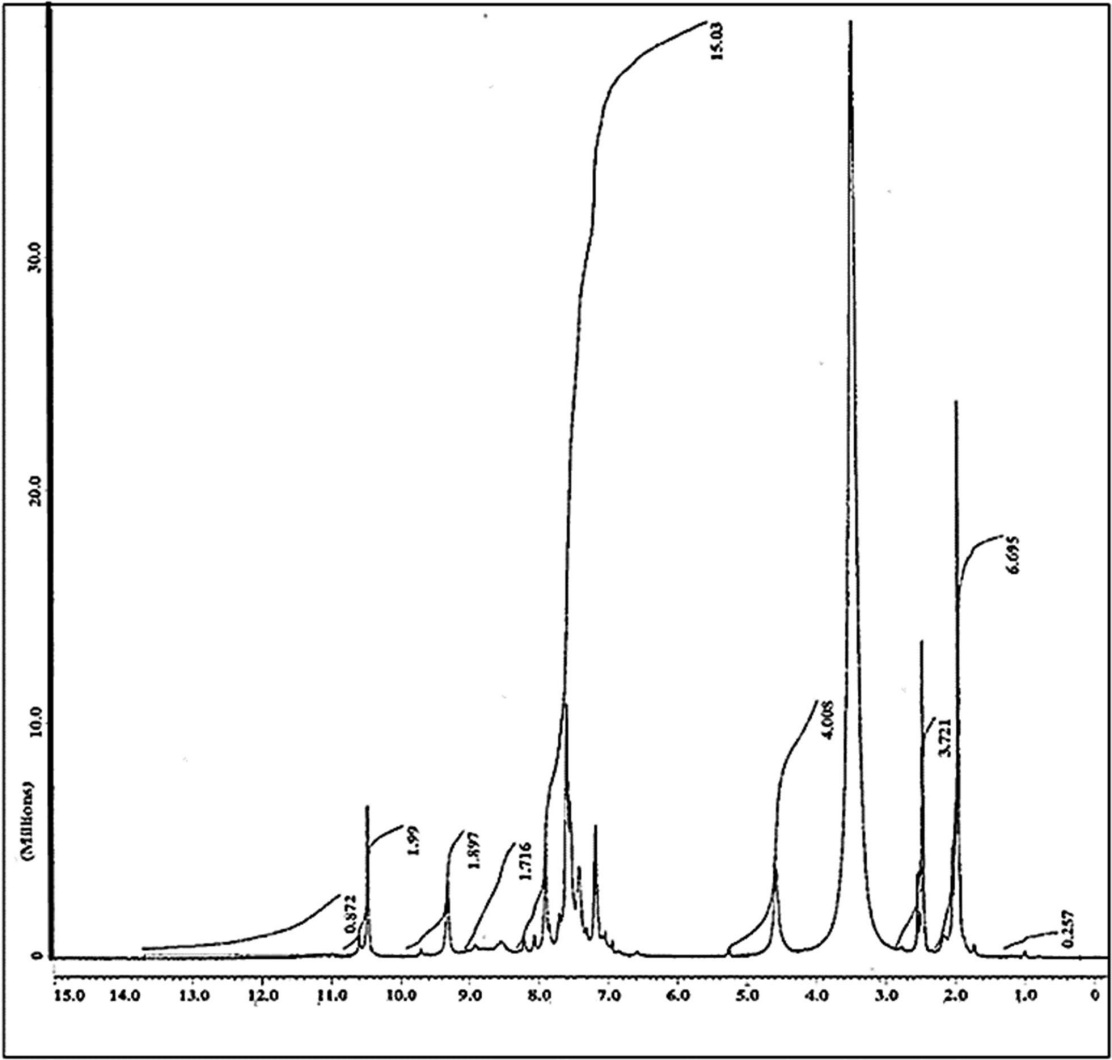
1H-NMR spectrum of the Cu(II) complex

On the other hand, the ^1^H-NMR spectra of the ligand gave six peaks at δ = 2.00, 4.50, (7.215–7.794, 8.511), (9.251–9.275) and 10.568 ppm assigned to CH_3_, CH_2_ groups, hydrogens of benzene and pyridine rings, –NH of [(CO)NHCH_2_] and -NH of** [**Ph.NH.(CO)CH_3_] groups respectively [57, 58], (Table 3, Fig. 4). All these peaks were shifted as a result of complex formation (Table 3, Fig. 5). The persistence of the hydrogens of the two -NH groups suggested that one –NH group acted as a neutral rather than an acidic group in complex formation, the other contributed in a H-bond. The band at δ = 4.581 ppm in the complex is attributed to the –OH groups of H_2_O molecule coordinated to Cu(II) [59] and assigned to hydrogen bonded OH groups of acidic character [60]. New peaks at δ = 10.9976, 11.1810, 11.5051 and 12.1042 ppm were observed as a result of H-bonding between –NH group and –C=O group and also between –NH group and the DMSO solvent used [61]. The band at 12.00 ppm can be attributed to the OH proton, indicating coordination of H_2_O molecule to the metal ion [62]. The optimized structures of the ligand and its metal complex were used to simulate their ^1^H NMR spectrum at DFT-B3LYP/6-31G(d, p) level using the Gauge-Including Atomic Orbital (GIAO) method [63]. The calculated ^1^H chemical shifts in DMSO, with Tetramethylsilane (TMS) as internal standard are given in Table 3 along with the experimental shifts. A good coherence between the theoretical and experimental chemical shifts for both ligand and its complex has been achieved.

**Table 3:** NMR chemical shifts of ligand and [CuLCl_2._H_2_O].2H_2_O

Ligand (L)			[CuLCl_2._H_2_O].2H_2_O	
	Experimental	Theoretical	Experimental	Theoretical
7-H	7.215–7.794	9.257	7.051–7.912	8.688
8-H		7.867		7.319
9-H		7.405		6.851
10-H		8.451		8.239
28-H	8.502 8.511	9.118	8.077 8.928	8.760
30-H		7.655		7.002
31-H		7.654		7.080
37-H		8.149		7.500
14-H	2.000	2.385	1.880	1.422
15-H		2.298		1.703
16-H		2.593		1.997
34-H	4.491	4.359	4.581	4.403
35-H	4.503	4.407		4.110
19-H	9.251, 9.263, 9.275	11.826	10.480	9.956
36-H	10.568	9.375	10.599	7.712

### Thermal analyses

Thermogravimetric analysis (TGA) of the Cu(II) complex was performed to get information about the thermal stability of the complex and to gauge whether the water molecules are in the ionic or the coordination sphere of the central metal ion. Heating rates were at 10 °C min^−1^ under nitrogen atmosphere with flow rate of 20 mL min^−1^ and weight losses were measured from ambient temperature up to 800 °C. Thermogravimetric analysis results agreed with the proposed structure.

**Fig. 6:**
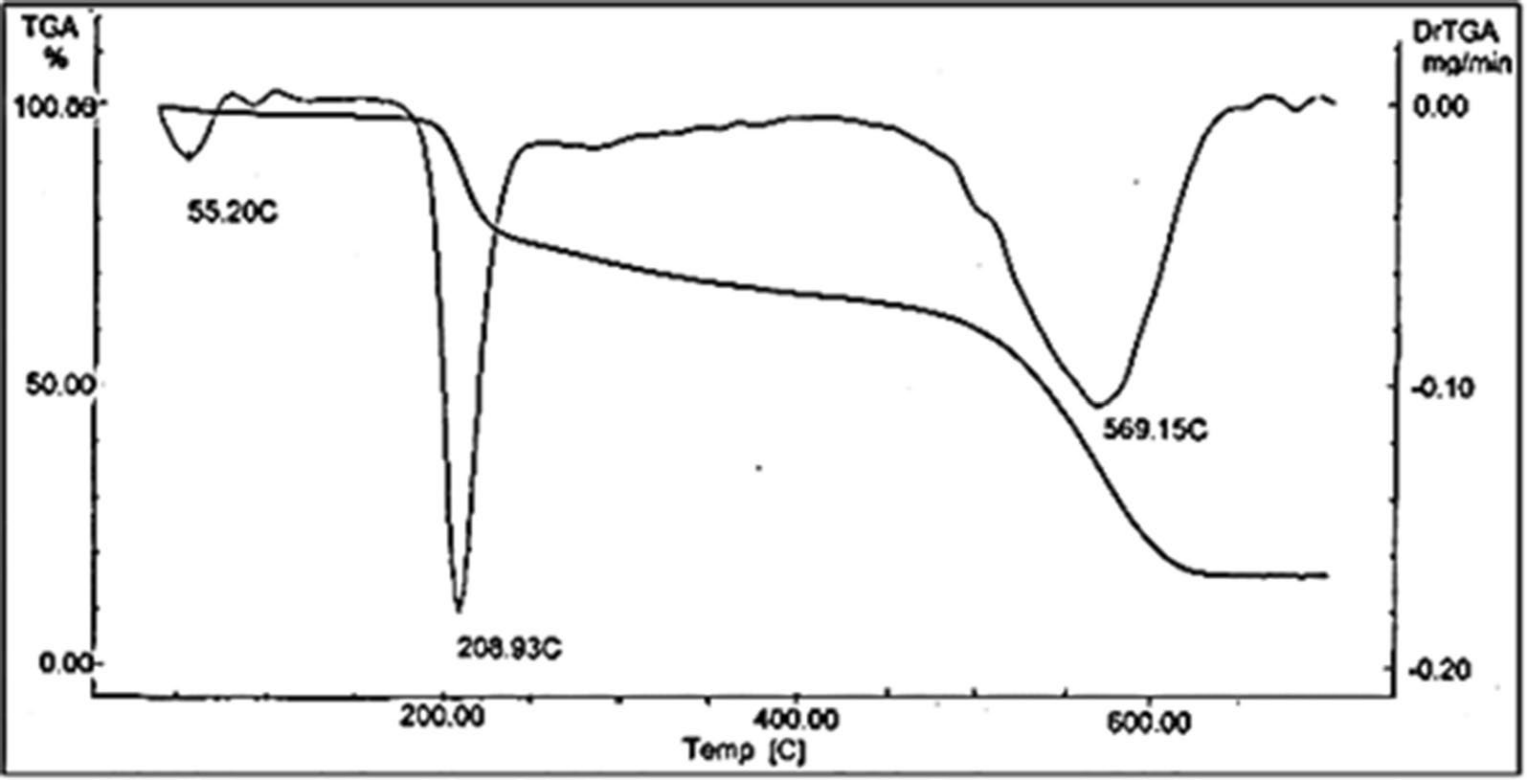
TGA and DTGA of the Cu(II) complex

The TGA of the [Cu.L.Cl_2_.H_2_O].2H_2_O complex (Fig. 6), showed the decomposition of the complex to occur in a three-step mechanism. It was mostly reported that the detached or non-coordinate water can be assigned in the TGA-temperature range from 30 °C to − 100 °C, while the coordinated water is mostly losing in between 100 °C and 150 °C [64–67]. It was observed that the first step between 40 and 252 °C is due to the decomposition of 3H_2_O (non-coordinated and coordinated) + NHCOCH_3_ moiety, (found: 23.455%; calculated: 23.067%). DTGA analysis described presence of the bands at (55.20, 95.00,130, 209 °C), in which water molecules were decayed and referred within these bands (55.20, 95.00 and 130 °C). The second step between 252 and 403 °C was attributed to the decomposition of Cl atom, (found: 7.643%; calculated: 7.312%) and the third step at the temperature range 403–659 °C assigned to the decomposition of Cl + (py-CH_2_NHCO.CO) + 4C atoms, (found: 50.314; calculated: 50.770%), with DTGA at 569°C, respectively and left with a residue of Cu + 2C atoms, (found: 18.328%; calculated: 18.031%).

**Fig. 7:**
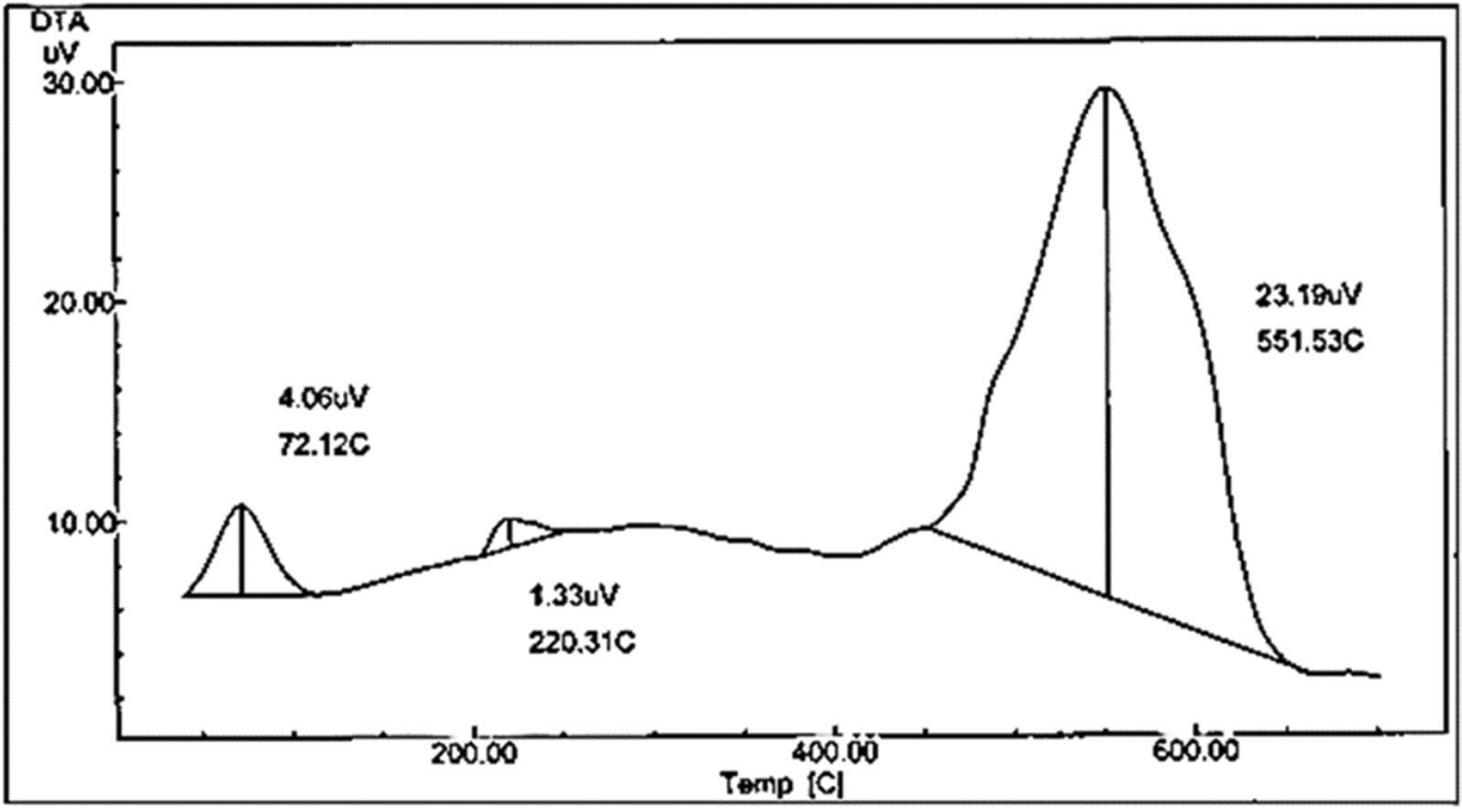
DTA of the Cu(II) complex

On the other hand, the DTA curve of the complex (Fig. 7), showed multiple exothermic peaks at 72.12 °C, 220.31 °C and 551.53 °C. Calculations were performed for the well resolved peaks only (the first and third). The thermodynamic parameters of decomposition processes of the complex, namely, activation energy (ΔEa^*^), enthalpy change (ΔH^*^) and entropy (ΔS^*^) were calculated by employing the Horowitz–Metzger equation [68] and are collected in Table 4. The order of chemical reactions (n) was calculated via the peak symmetry method by Kissinger [69] and the asymmetry of the peak, S, was calculated as follows:

**Table 4:** Thermodynamic parameters of the Cu(II) complex based on the DTA curve

peak	∆E KJ/mol	n	α	T_m_	Z	∆S^*^ KJ/mol K	∆H^*^ KJ/mol	∆G^*^ KJ/mol
a	127.204	1.38	0.571	344	2.525	− 0.238	− 82.009	− 0.138
c	144.879	1.29	0.583	555	1.080	− 0.253	− 209.025	− 68.585

$$n = 1.26{\left( {\frac{a}{b}} \right)^{1/2}}$$

$$S=0.63 {n}^{2}$$

The value of the decomposed substance fraction, ${\alpha }_{m}$, at the moment of maximum development of the reaction (with $T= {T}_{m}$) was determined from the relation [70]

$$\left( {1 - {\alpha _m}} \right) = {n^{\left[ {1 - \left( {1/n} \right)} \right]}}$$

The values of collision factor, Z, can be obtained by making the use of the relation:

$$Z= \left(\frac{{{E}_{a}}^{*}}{R{T}_{m}}\right)\beta \text{exp} \left(\frac{{{E}_{a}}^{*}}{R{{T}_{m}}^{2}}\right)$$

where $R$ = the molar gas constant, $\beta$ is the heating rate (Ks^−1^), ${T}_{m}$ is the peak temperature.

The entropies of activation,$\Delta {S}^{*}$, are calculated from the equation [71]:

$$Z=\left(\frac{k{T}_{m}}{h}\right)\text{exp}\left(\frac{\Delta {S}^{*}}{R}\right)$$

where $k$ is Boltzmann constant and $h$ is Planck’s constant.

The change in enthalpy $\Delta {H}^{*}$, taking place at any peak temperature, ${T}_{m}$, can be given by the following equation:

$$\Delta {S}^{*}= \frac{\Delta {H}^{*}, }{{T}_{m}}$$

**Fig. 8:**
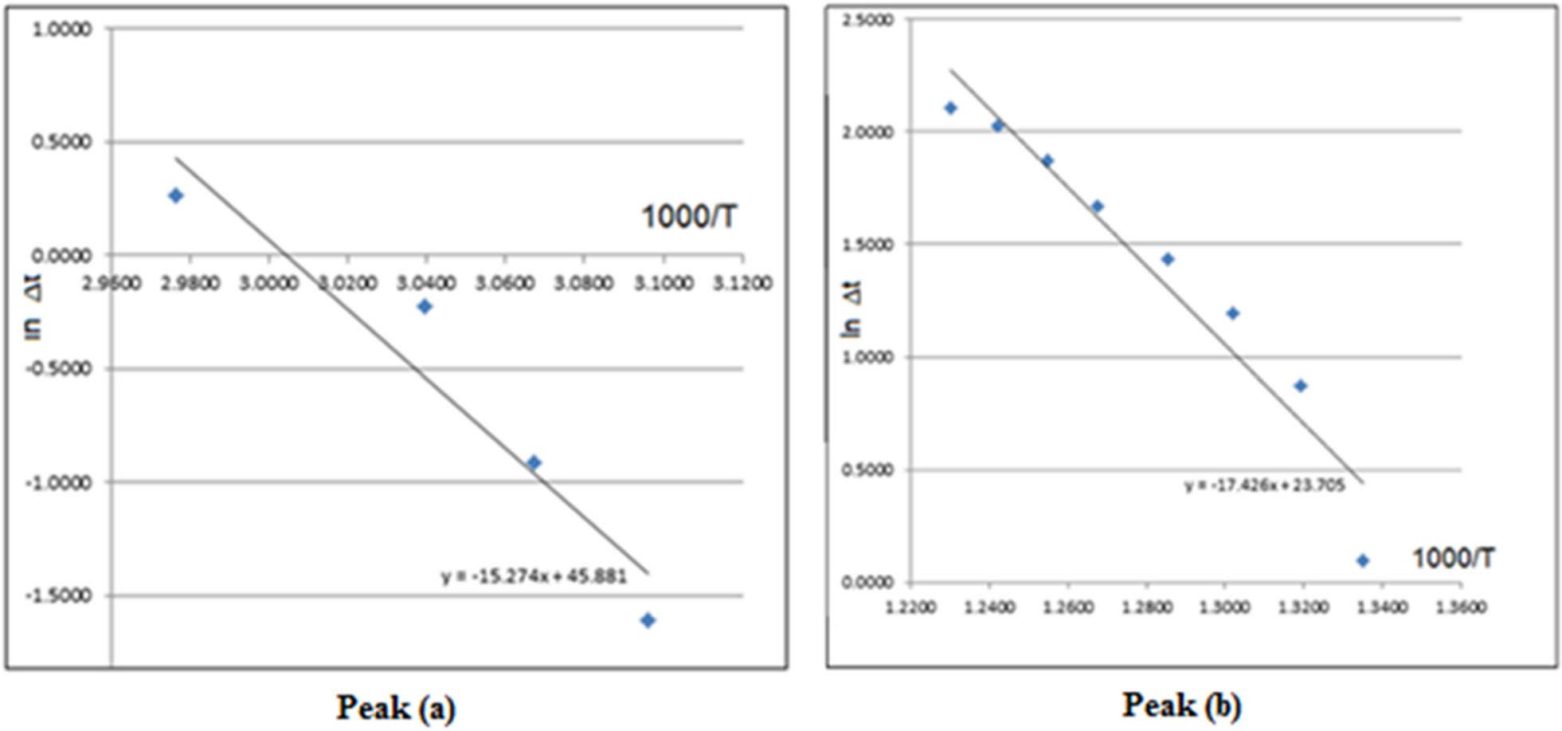
ln Δt versus 1000/T plots from DTA curve of the Cu(II) complex

Based on least square calculations, the *ln* Δt versus 1000/T plot for the complex, for each peak (Fig. 8), gave straight lines from which the activation energies were calculated according to the method of Piloyan et al. [72]. The slope is of Arrhenius type and equals − E_a_^*^ / R.

According to the data obtained from the DTA curve, some conclusions can be achieved as follows: (i) the appearance of fractional orders (n) suggested that the reaction proceeds via complicated mechanisms but approaching first order. (ii) The change of entropy values, ΔS^*^, for the complex, (Table 4), are nearly of the same magnitude (− 0.234 and − 0.242) kJ K^−1^ mol^−1^ indicating that the transition states are more ordered, thus in a less random molecular configuration than the reactants [73] (iii) The values of ∆E^*^ were 127.204 and 144.879 kJ/mol, and ∆H^*^ values were − 82.00 and − 209.02 kJ/mol for the first and third peaks, respectively.

The negative values of ∆H^*^ indicated the exothermic nature of the metal–ligand interaction. Negative values of ∆G^*^ showed spontaneous formation of the complexes [74]. The entropy change (∆S^*^), which is a measure of disorder or randomness in the system, are negative, indicating that complex formation is favorable [5]. The enthalpy change is large and is enough to overcome the entropy change which favors the spontaneous formation of the complex [75, 76].

### Experimental UV–vis and ESR spectra

Experimental electronic absorption and ESR spectra of the Cu(II) complex were recorded in order to understand the spatial arrangement of the metal ion around the ligand. Intense electronic spectral bands for the ligand were observed between 243 and 371 nm assigned to π→π* transition originating in the phenyl ring and C=O groups [77], the bands at 440 and 475 nm are attributed to n→π* transitions originating in the –NH-CO chromophores. In the spectrum of the Cu(II) complex, these bands were shifted to lower energies as a result of complex formation.

The nujol mull electronic spectrum of the copper(II) complex showed a broad d-d band in the 540 nm region (18,518 cm^−1^) which can be assigned to 2T2g → 2Eg transition of an octahedral geometry [33]. Though under the influence of the tetragonal distortion, the 2Eg and 2T2g states of the octahedral Cu(II) ion (d9) split, and three transitions 2B1g→2Eg, 2B1g →2B2g, and 2B1g → 2A1g are expected [78–80], but their very close energies makes them appear in the form of one broad band. However, in ethanol solvent, these bands were detected at 540 nm (18,518 cm^−1^), 656nm (15,244 cm^−1^) and 715 nm (13,986 cm^−1^), which are in good agreement with those reported for a distorted octahedral geometry around Cu(II) ion [76]. The magnetic moment value of 1.92 BM together with the electronic data suggested the existence of the Cu(II) complex in a monomeric form with a distorted octahedral environment with dx2-y2 ground state [81].

**Fig. 9:**
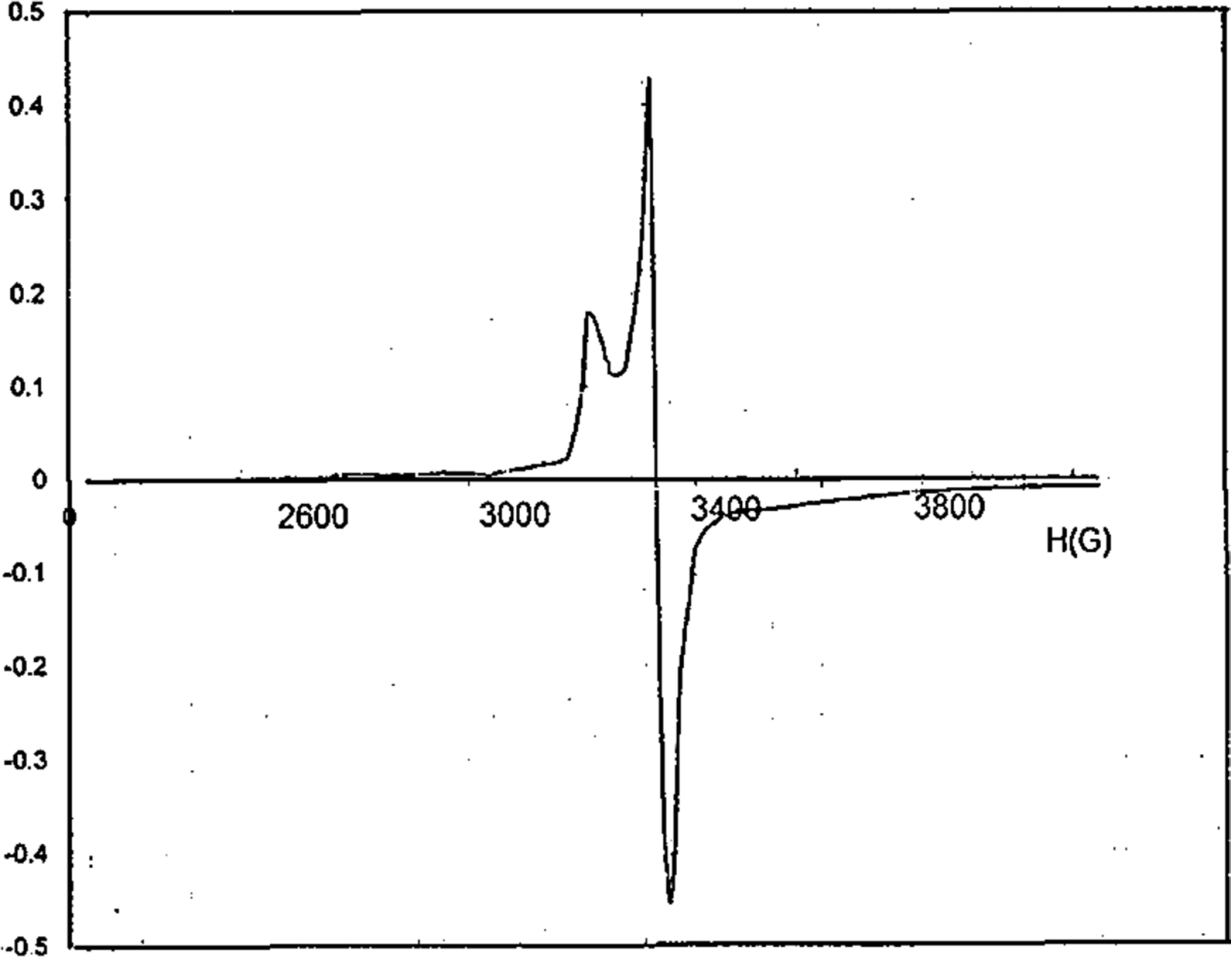
ESR spectrum of the Cu(II) complex

The X-band ESR spectrum of the copper(II) complex in the polycrystalline state was recorded at 298 K and studied in the 2300–4000 G region (Fig. 9), to elucidate the geometry and degree of covalency of the metal–ligand bond or environment around the metal ion. The g-factors were measured using DPPH as standard. The spectrum exhibited an axial behavior with g// (2.107) and g┴ (2.022) values. The geometric parameter “G” which is a measure of the exchange interaction between the copper(II) centers in the polycrystalline compound was calculated using the equation: G = (g// − 2.0023) / (g┴ − 2.0023) [81]. It was found that g// > g┴ > 2.0023 and G = 4.707. According to Hathaway [82], if G > 4.0, the local tetragonal axes are aligned parallel or only slightly misaligned. If G < 4.0, significant exchange coupling is present and the misalignment is appreciable. The g- and G-values were consistent with a dx2-y2 ground state in a monomeric distorted octahedral structure [83].

### Electronic absorption spectra (TD-DFT)

**Fig. 10:**
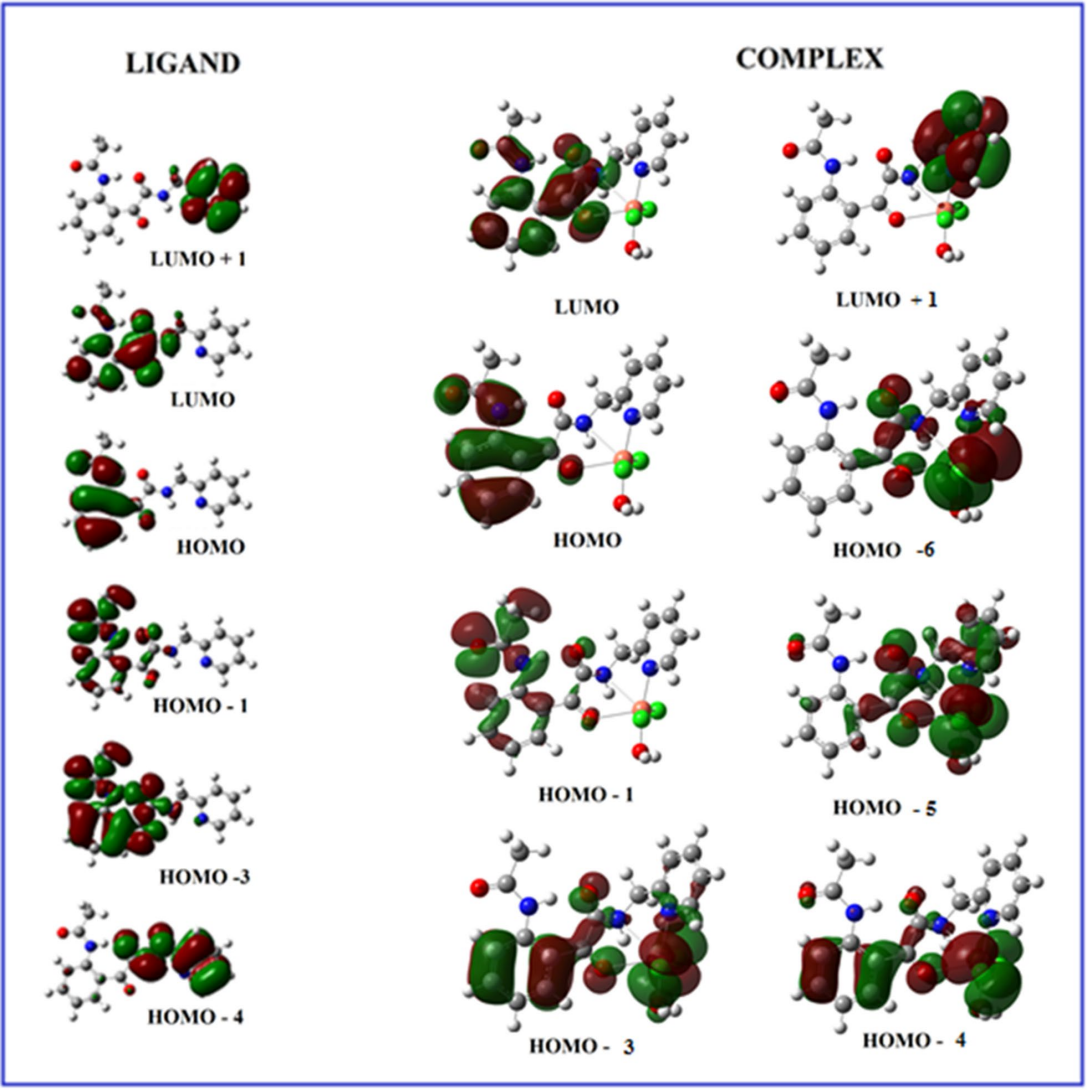
Molecular orbitals of ligand and its Cu (II) complex involved in electronic transitions

The highest occupied molecular orbital, HOMO (electron donor orbital) and lowest unoccupied molecular orbital (LUMO) (electron acceptor orbital) are well known quantum chemical descriptors. The energy gap between HOMO and LUMO is used as an indicator for chemical reactivity and kinetic stability of a molecule. A molecule is said to be soft with a high chemical reactivity, if it has a small HOMO–LUMO gap [84, 85]. In order to have a better insight and further understanding of the structures of ligand (L) and its metal complex, their electronic structures have been probed theoretically. The energy of the HOMO and LUMO orbitals and their orbital energy gap are calculated using the TD-DFT method, and the pictorial illustrations of these frontier orbitals for both the ligand and its Cu(II) complex are shown in Fig. 10. Theoretical vertical excitation energies (E), oscillator strengths (f) and the corresponding absorption wavelengths (λ) are collected in Table 5.

**Table 5:** Experimental and calculated absorption wavelength λ (nm), excitation energies E (eV), absorbance values and oscillator strengths (*f*) of ligand and its complex

Experimental λ (nm)	TD-DFT λ (nm)		E (eV)	*f*
Ligand				
440	403.31	HOMO → LUMO (91%)	3.0742	0.0577
371	380.19	H-3 → LUMO (41%)	3.2611	0.0140
315	338.70	H-1 → LUMO (88%)	3.6606	0.0058
300	320.94	H-3 → LUMO (49%), H-2 → LUMO (47%)	3.8631	0.1274
285	295.38	H-4 → LUMO (96%)	4.1974	0.0499
270	279.10	H → LUMO + 1 (100%)	4.4423	0.0018
Complex				
720	816.80	H-5(B) → LUMO(B) (19%); H-7(B) → LUMO(B) (16%),	1.5179	0.0011
656	601.19	H-6(B) → LUMO(B) (50%)	2.0623	0.0022
540	565.28	H-7(B) → LUMO(B) (29%), H-6(B) → LUMO(B) (21%)	2.1933	0.0031
	519.56 509.95	H-1(B) → LUMO(B) (45%) H-1(B)) → LUMO(B) (48%)	2.3863 2.4313	0.0097 0.0121

*H* HOMO, *B* beta orbital

**Fig. 11:**
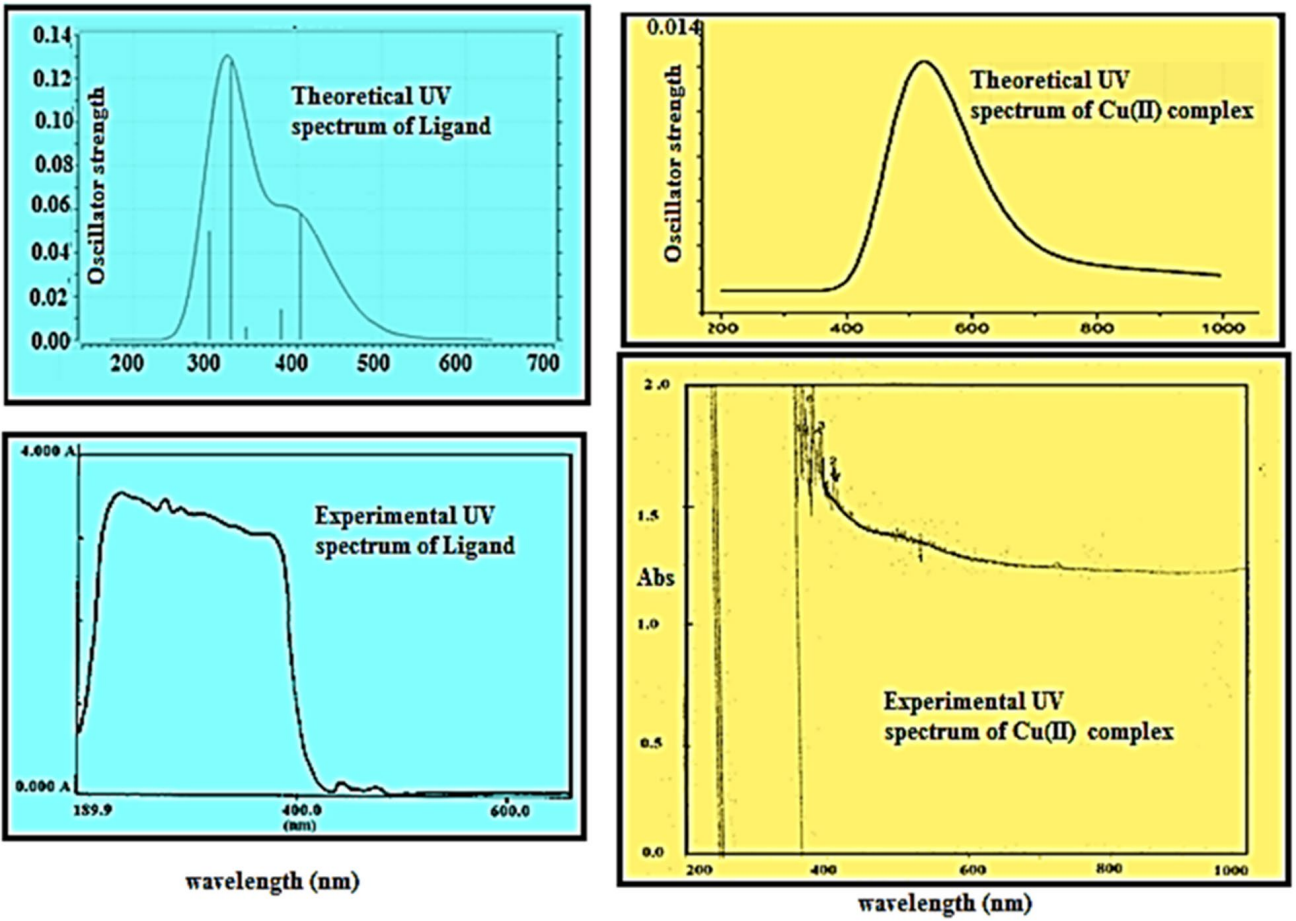
Experimental and theoretical UV Spectra of the ligand and its Cu(II) complex

**Fig. 12:**
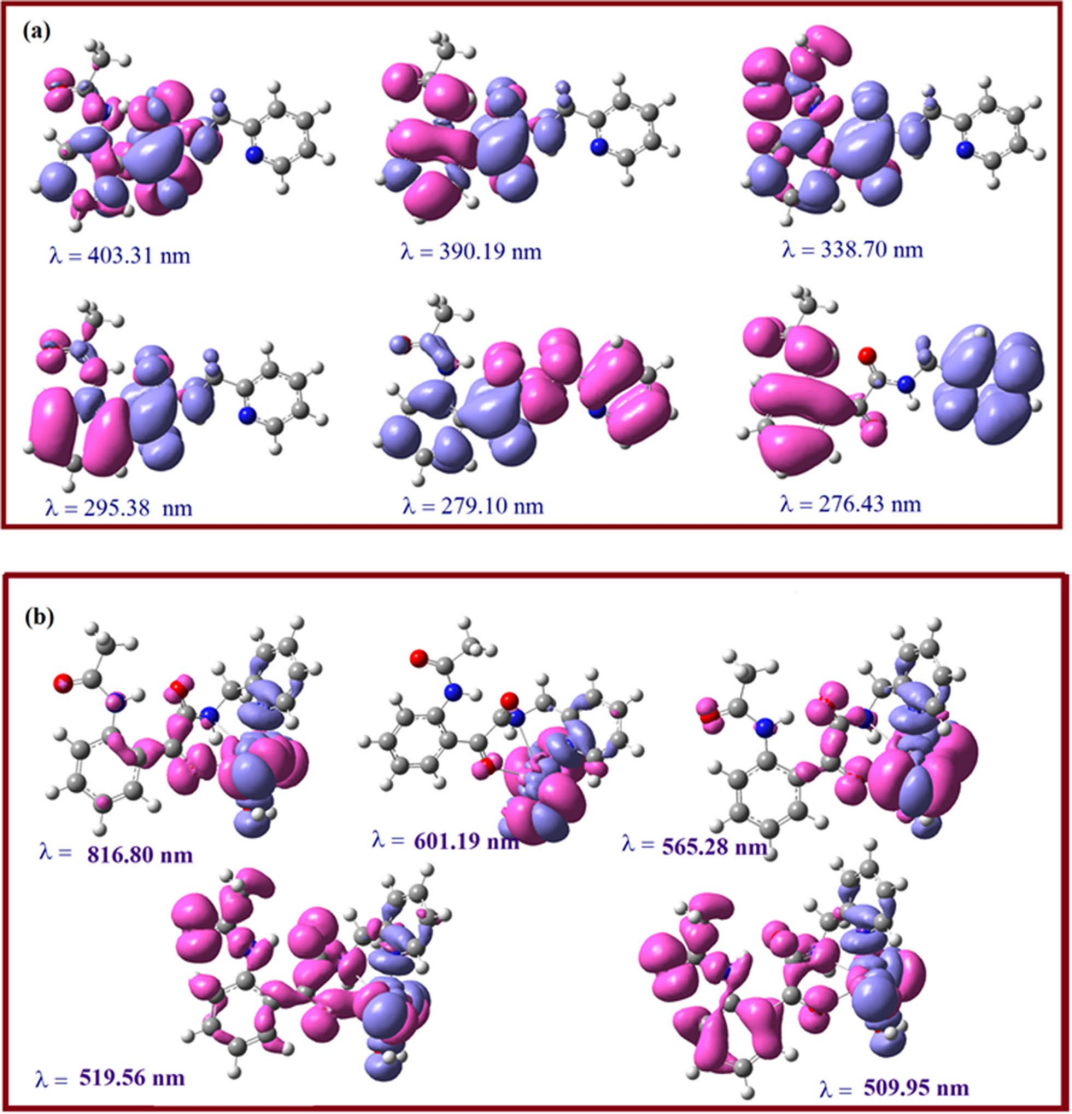
The total electron density difference between ground and excited states. **a** Ligand. **b** Complex

In the ligand the calculations predict one intense electronic transition at 295.38 nm with an oscillator strength 0.0499, in agreement with the experimental value shown in Fig. 11. This electronic absorption mainly corresponds to the transition from the molecular orbital HOMO(H)-4 to LUMO(L). The other two peaks at 279.10 and 403.31 nm in the calculated UV spectrum in gas phase arises mainly due to the electronic transition given by H → L + 1 and H → L respectively. In the metal complex [Cu.L.Cl_2_.H_2_O].2H_2_O the highest peak with oscillator strength 0.0121 appears at 509.95 nm in line with the experimental peak at 540 nm. As the spin of the complex is 1/2, this corresponds to transition between beta orbitals H-1 → L. The HOMO–LUMO energy gap (∆E) for the ligand and complex are calculated to be 3.68660 and 3.72378 eV respectively. To analyze the charge transfer characteristics of the electronic excitation, the net electron density difference between ground and excited states have been plotted for ligand as well as complex (Fig. 12). In Fig. 12, the pink color represents where the electrons are coming from, and the light blue color represents where the electrons are going. The electron density difference plot of the ligand indicates the large pink lobes around the oxygen atoms and blue antibonding orbitals around phenyl ring thus the transition at λ = 403.31 nm to be n → π* type. The transition at λ = 279.10 nm shows the transfer of electron charge density from pyridin-2-ylmethyl)acetamide moiety to acetamidophenyl moiety and to be of π → π* type. In Cu (II) complex the major charge density is transferred around metal binding site for λ = 540 nm.

### Antimicrobial and antifungal activities

The fate of novel bioactive substances in terms of therapy is heavily influenced by chelation and were reported as a significant antimicrobial agent [86]. The antibacterial and antifungal activities of the synthesized Cu(II) complex were screened against a Gram-negative (*E. coli*) and a Gram-positive (*S. aureus*) bacteria in addition to *A. flavus*, and *C. albicans* fungi. The Cu(II) complex was found to have considerable effect on both gram positive and gram negative bacteria as well as on *Aspergillus flavus* and *Candida albicans* fungus as given in Table 6.

**Table 6:** Inhibition zone diameter values in mm/mg for the Cu(II) complex

Compound	Inhibition zone diameter (mm/mg sample)			
	*Escherichia coli* (G^−^)	*Staphylococcus aureus* (G^+^)	*Aspergillus flavus* (Fungus)	*Candida albicans* (Fungus)
Control: DMSO	0.0	0.0	0.0	0.0
Standard: Tetracycline Antibacterial agent	32	30	–	–
Standard: Amphotericin B Antifungal agent	–	–	19	20
[Cu.L.Cl_2_.H_2_O].2H_2_O	19	18	11	11

### Molecular docking simulation with insulin-like growth factor-1 receptor (IGF-1R)

#### Data processing and prediction

**Fig. 13:**
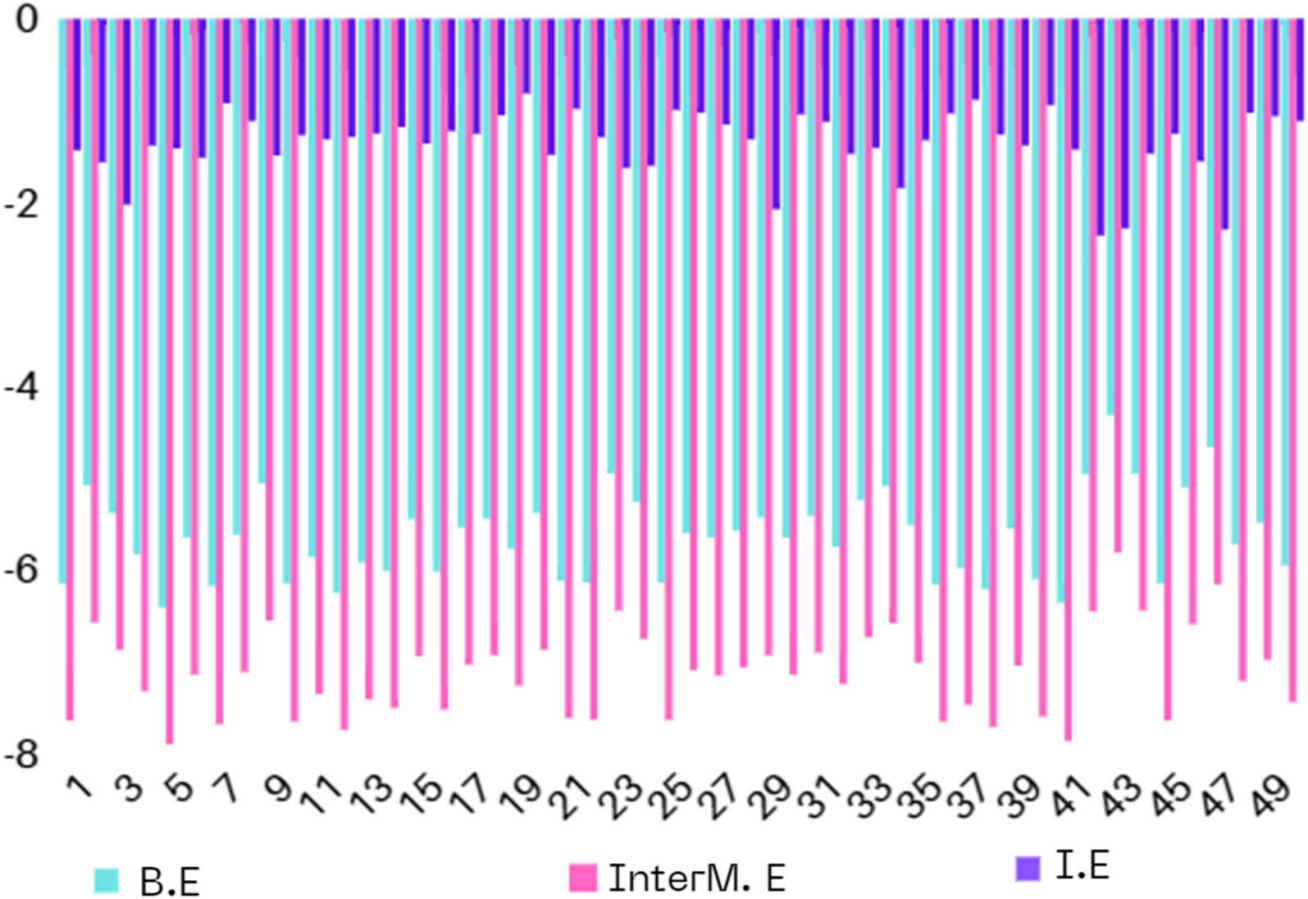
Correlation between score energies, B.E, InterM.E and I.E, resulted from 50 selected conformations for the synthesized ligand (L) bound with IGF-1R target

Molecular docking was processed using Autodock accurate settings involving 50 conformational structures to be analyzed according to binding energy (B.E), intermolecular energy (InterM.E) and internal energy (I.E) with inhibition constant evaluating values [87]. 5FXR protein target was prepared and become ready as a receptor input for the synthesized ligand. the conformational structures are present in Table 7 investigating the most important analyzable energies that mainly help in the ligand-target stability. Figure 13 shows a correlation between the different types of energies come from docking analysis of 50 conformational ligand structures.

**Table 7:** The conformational dockings of the synthesized ligand (L) with IGF-1R target (ID:5FXR) exhibiting different score energies

Conformational step	5FXR			
	Binding energy	Intermolecular energy	Internal energy	Inhibition constant
1	− 6.14	− 7.63	− 1.43	31.39
2	− 5.07	− 6.56	− 1.56	193.05
3	− 5.37	− 6.86	− 2.02	115.98
4	− 5.82	− 7.31	− 1.38	54.28
5	− 6.40	− 7.89	− 1.41	20.29
6	− 5.64	− 7.13	− 1.51	73.72
7	− 6.17	− 7.67	− 0.92	29.83
8	− 5.61	− 7.10	− 1.11	77.69
9	− 5.05	− 6.54	− 1.49	199.42
10	− 6.14	− 7.64	− 1.27	31.34
11	− 5.85	− 7.34	− 1.31	51.72
12	− 6.24	− 7.73	− 1.29	26.67
13	− 5.91	− 7.40	− 1.25	46.29
14	− 6.00	− 7.49	− 1.18	40.07
15	− 5.44	− 6.93	− 1.36	102.69
16	− 6.01	− 7.51	− 1.22	39.04
17	− 5.53	− 7.02	− 1.25	88.11
18	− 5.43	− 6.92	− 1.05	104.77
19	− 5.76	− 7.25	− 0.81	60.30
20	− 5.37	− 6.86	− 1.48	115.24
21	− 6.11	− 7.60	− 0.98	33.20
22	− 6.13	− 7.62	− 1.29	32.35
23	− 4.94	− 6.43	− 1.62	241.19
24	− 5.25	− 6.74	− 1.6	142.89
25	− 6.13	− 7.62	− 0.99	31.97
26	− 5.59	− 7.08	− 1.02	79.77
27	− 5.64	− 7.14	− 1.15	72.97
28	− 5.56	− 7.05	− 1.31	84.58
29	− 5.42	− 6.92	− 2.07	105.56
30	− 5.64	− 7.13	− 1.04	73.52
31	− 5.40	− 6.89	− 1.12	109.71
32	− 5.74	− 7.23	− 1.47	61.92
33	− 5.23	− 6.72	− 1.4	146.19
34	− 5.08	− 6.57	− 1.84	189.26
35	− 5.50	− 7.00	− 1.32	92.35
36	− 6.15	− 7.64	− 1.03	31.15
37	− 5.97	− 7.46	− 0.88	42.23
38	− 6.20	− 7.70	− 1.26	28.32
39	− 5.54	− 7.03	− 1.38	86.87
40	− 6.09	− 7.59	− 0.94	34.13
41	− 6.35	− 7.85	− 1.42	21.97
42	− 4.95	− 6.44	− 2.36	234.47
43	− 4.31	− 5.80	− 2.28	698.81
44	− 4.94	− 6.43	− 1.47	240.16
45	− 6.14	− 7.63	− 1.25	31.65
46	− 5.09	− 6.58	− 1.55	186.87
47	− 4.66	− 6.15	− 2.29	381.84
48	− 5.71	− 7.20	− 1.02	64.88
49	− 5.48	− 6.97	− 1.06	96.94
50	− 5.94	− 7.43	− 1.11	44.01

#### Conformational docking analysis

**Fig. 14:**
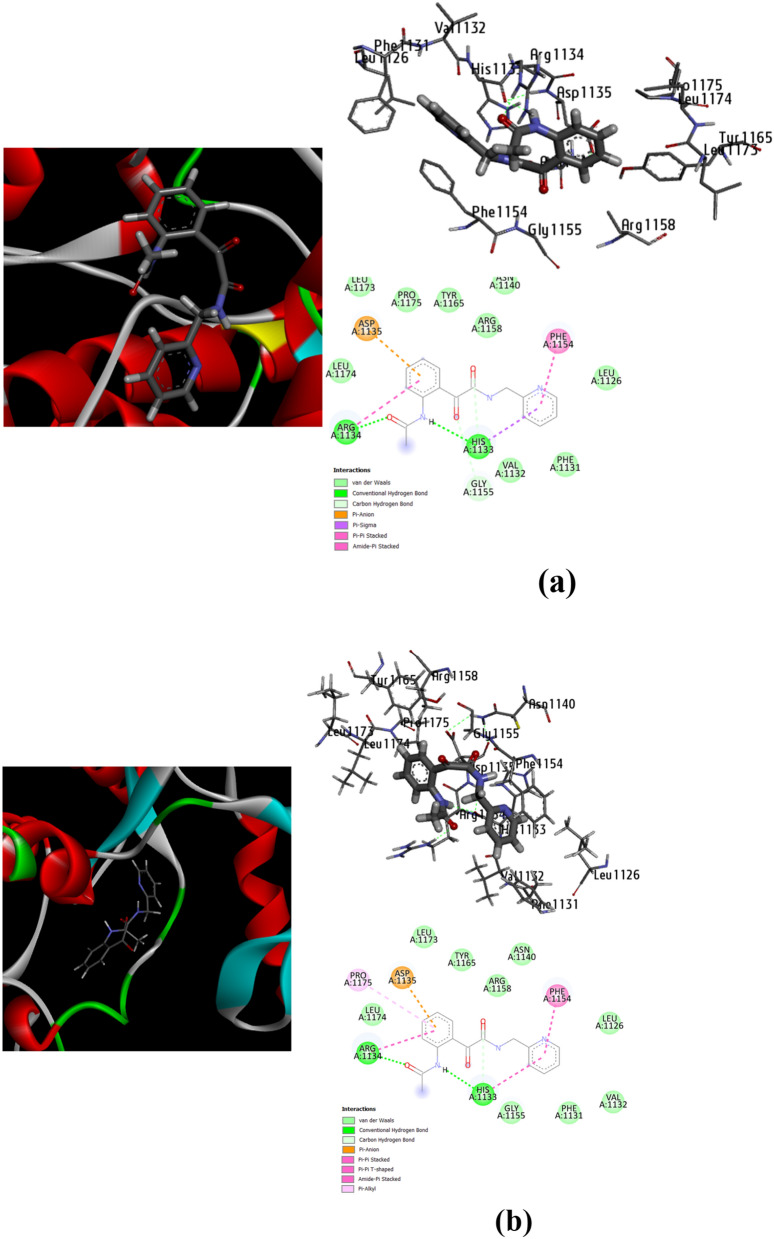
Conformers of complex-ligand interactions for the more potent poses 5, **(a)** and 41, **(b)**

**Fig. 15:**
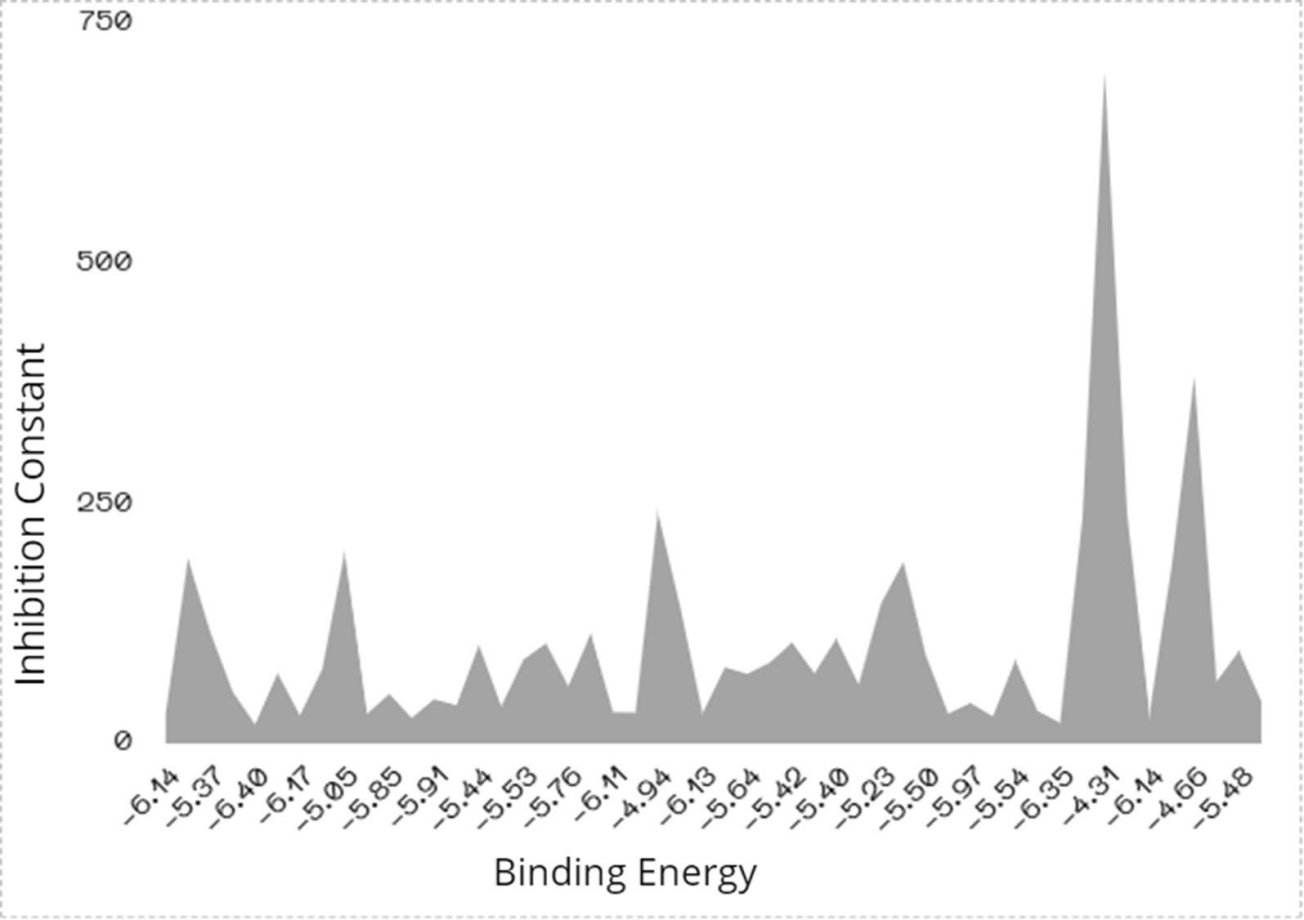
Correlation between binding energy and inhibition constant of each conformational structure

As shown from Fig. 13, the preferable energetic pose is located in conformational structure 5 and 41 (B.E = − 6.40 kcal/mol and − 6.35 kcal/mol) (Table 7). The types of non-covalent interactions mainly identify the stability of each conformational structure. Figure 14 represents the types of complex-ligand interactions for the poses of 5 and 41. In Fig. 14a, amino acids of HIS 1133.A, ARG 1134.A are interacted with the ligand by conventional H-bonding. Additionally, GLY 1155.A is interacted by carbon-H bond. These types of H-bonds mainly affect on the inhibitory ability of the ligand towards the target. Also, the π-electrons help in bio-complex stability and then inhibition efficiency. There are a number of π-interactions such π-anion, π-sigma, π–π stacked and amide- π stacked, that participate in selection of pose stabilization, also van der Waals interaction is present profusely around the conformational complexed structure. Furthermore, the complexed pose of 41 (Fig. 14b) shows similar interaction types mainly present in HIS1133.A and ARG 1134.A showing conventional H-bonding and ARG 1134.A showing carbon-H bonding. Other non-covalent interactions are prevalent around the bio-complex and slightly differ from pose 5 in presence of π-alkyl instead of π-sigma and presence additional π-π T-shaped interaction. Figure 15 correlates the binding energy with the inhibition constant values.

## Conclusions

The Cu(II) complex, synthesized using the ligand 2-(2- acetamidophenyl)-2-oxo-*N*-(pyridin-2-ylmethyl)acetamide, was investigated using elemental, spectral, magnetic measurement, molar conductivity, and thermal analysis. The magnetic moment value together with the electronic spectrum suggested the existence of the Cu(II) complex in a monomeric form with a distorted octahedral environment with d_x_^2^_-y_^2^ ground state. Computational studies revealed that the HOMO positions of the complexes are localized mostly around the acetamidophenyl group of the ligand while the LUMO is distributed around the metal centre. The plots of total electron density difference between ground and excited states in ligand clearly illustrates the transition corresponding to λ = 403.31 nm and λ = 279.10 nm to be respectively of n → π* and π → π* type. The synthesized complex was screened for its antibacterial activity against S. aureus and E. coli and for antifungal activity against *A. flavus* and *C. albicans* and found to have considerable activity against these. Conformational analysis illustrated presence of most stable conformational structure of the synthesized ligand around Insulin-like growth factor 1 receptor (ID: 5FXR).

## Data Availability

The datasets used and/or analyzed during the current study are available from the corresponding author on reasonable request.
